# Resection of brain lesions with a neuroendoscopic ultrasonic aspirator — a systematic literature review

**DOI:** 10.1007/s10143-022-01837-w

**Published:** 2022-07-27

**Authors:** Florian Ebel, Ladina Greuter, Raphael Guzman, Jehuda Soleman

**Affiliations:** 1 Department of Neurosurgery, University Hospital of Basel, Spitalstrasse 21, 4031 Basel, Switzerland; 2 Department of Pediatric Neurosurgery, University Children’s Hospital of Basel, Basel, Switzerland; 3 Faculty of Medicine, University of Basel, Basel, Switzerland

**Keywords:** Neuroendoscopy, Oncology, Ultrasonic aspirator, Surgical technique

## Abstract

The development of minimally invasive neuroendoscopy has advanced in recent years. The introduction of the neuroendoscopic ultrasonic aspirator (NUA) increased the treatment spectrum of neuroendoscopy. This review aimed to present a systematic overview of the extent of resection, lesion characteristics, technical aspects, complications, and clinical outcomes related to using the NUA. Articles were identified by searching the PubMed/Medline, Embase, and Web of Science database through June 2022 with restriction to the last 20 years. We included case series, case reports, clinical trials, controlled clinical trials, meta-analyses, randomized controlled trials, reviews, and systematic reviews written in English. Studies reporting on endonasal approach or hematoma evacuation using the NUA were excluded. The references of the identified studies were reviewed as well. Nine full-text articles were included in the analysis, with a total of 40 patients who underwent surgery for a brain tumor using NUA. The most common underlying pathology treated by NUA was colloid cyst (17.5%), pilocytic astrocytoma (12.5%), subependymal giant cell astrocytoma (7.5%), subependymoma (7.5%), and craniopharyngioma (7.5%). Complete or near-total resection was achieved in 62.5%. The most frequently reported postoperative complication was secondary hydrocephalus (10%), meningitis/-encephalitis (7.5%), cognitive impairment (7.5%), and subdural hygroma (7.5%). In one case (2.5%), surgery-related death occurred due to a severe course of meningoencephalitis. According to the preliminary data, NUA seems to be a safe and efficient minimally invasive alternative to conventional microscopic resection of brain tumors. Further studies to investigate advantages and disadvantages of using the NUA are needed.

**Supplementary Information:**

The online version contains supplementary material available at 10.1007/s10143-022-01837-w.

## Introduction

Neuroendoscopy has become a valuable and important instrument for treating various neurosurgical pathologies in recent decades [1]. Before introducing neuroendoscopic ultrasonic aspirators (NUA), and other suction devices like the NICO Myriad (NICO Corp., Indianapolis, USA) and Artemis (Penumbra, Alameda, USA), the fields of application for neuroendoscopy were limited to the treatment of hydrocephalus, biopsies, or partial resection of brain lesions [2]. The first ultrasonic aspirator (UA) application for open neurosurgical procedures was described in 1978 [3]. There were numerous modifications and further improvements to the UA in the following years.

Initially, animal experiments were carried out, which showed good and safe handling, whereon NUA was used for the first time in five clinical cases in 2008 [2]. The technical possibilities are currently still limited, but NUA has led to progress in the endoscopic resection of intraventricular brain lesions.

To the best of our knowledge, this is the first systematic literature review focusing on the use of NUA for the resection of brain lesions. This review aimed to present a systematic overview of the extent of resection, tumor characteristics, technical aspects, complications, and clinical outcomes related to using NUA.

## Methods

The systematic literature search was carried out following the updated PRISMA Guidelines 2020 [4]. The literature database PubMed/Medline, Embase, and Web of Science was searched, and appropriate clinical studies published before 14.06.2022 were identified.

**Fig. 1:**
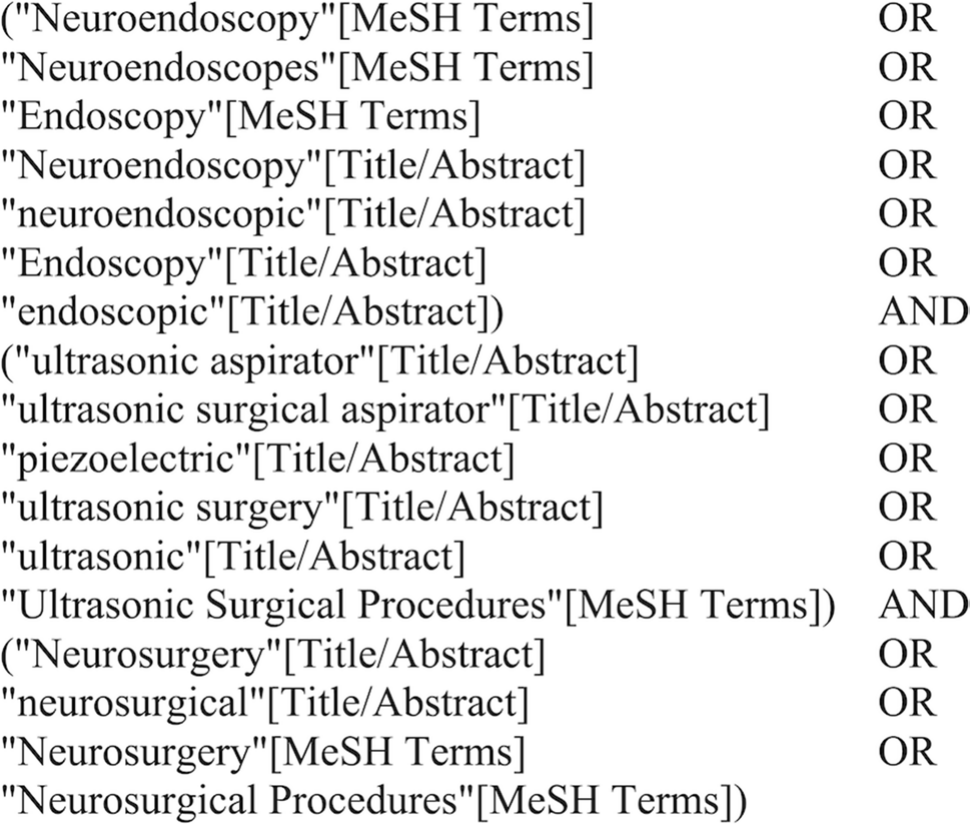
Search string used for PubMed, Embase, and Web of Science database search

We used a search term with the keywords “neuroendoscopy & endoscopy,” “ultrasonic aspirator,” and “neurosurgery” in the PubMed/Medline, Embase, and Web of Science databases with restrictions to English language (Fig. 1). We included case series, case reports, clinical trials, controlled clinical trials, meta-analyses, randomized controlled trials, reviews, and systematic reviews that reported cases in which brain tumors were resected neuroendoscopically by NUA. Studies reporting on the endonasal use or hematoma evacuation using the NUA were excluded.

Two authors (F.E. and J.S.) independently assessed all results for eligibility. Where consensus opinion could not be reached, a third researcher was to be consulted (R.G.). First, all duplicates were excluded. Then, the titles and afterwards the abstracts were analyzed. The remaining studies were then included in the full-text analysis. All included full-text studies underwent independent quality assessment (F.E. and J.S.) using the Joanna Briggs Institute (JBI) Critical Appraisal Checklist [5]. All nine included full-text studies met the quality criteria of the JBI checklist and none had to be excluded.

As outcome parameters, we primarily analyzed the extent of resection (EOR) and secondarily the various tumor characteristics, and intraoperative and postoperative complications, as well as the recurrence rate at the resection site, clinical outcome during follow-up, and technical aspects. In the article of Spennato et al., only the tumor volume (cm^3^) was reported. To compare it with the other included studies, we approximately converted the volume (*v*) into a diameter (*d*) using the formula $(d =\sqrt[3]{2*v}$). To simplify the data presentation, we divided the EOR into three groups and defined them as follows: gross total resection (GTR) as a complete resection without evidence of a residual tumor on postoperative imaging, near total resection (NTR) with a minimal tumor remnant (> 95% resection extent), and subtotal resection (STR) if larger remnants were left in situ during surgery.

### Statistical analysis

All analyses were done using the SPSS Software (Version 28, IBM Corp., New York, USA). Univariate analysis was done using the Fisher exact or chi-square test for categorical data and Mann–Whitney *U* or the Kruskal–Wallis test for continuous data. A value of *P* < 0.05 was considered significant.

## Results

**Fig. 2:**
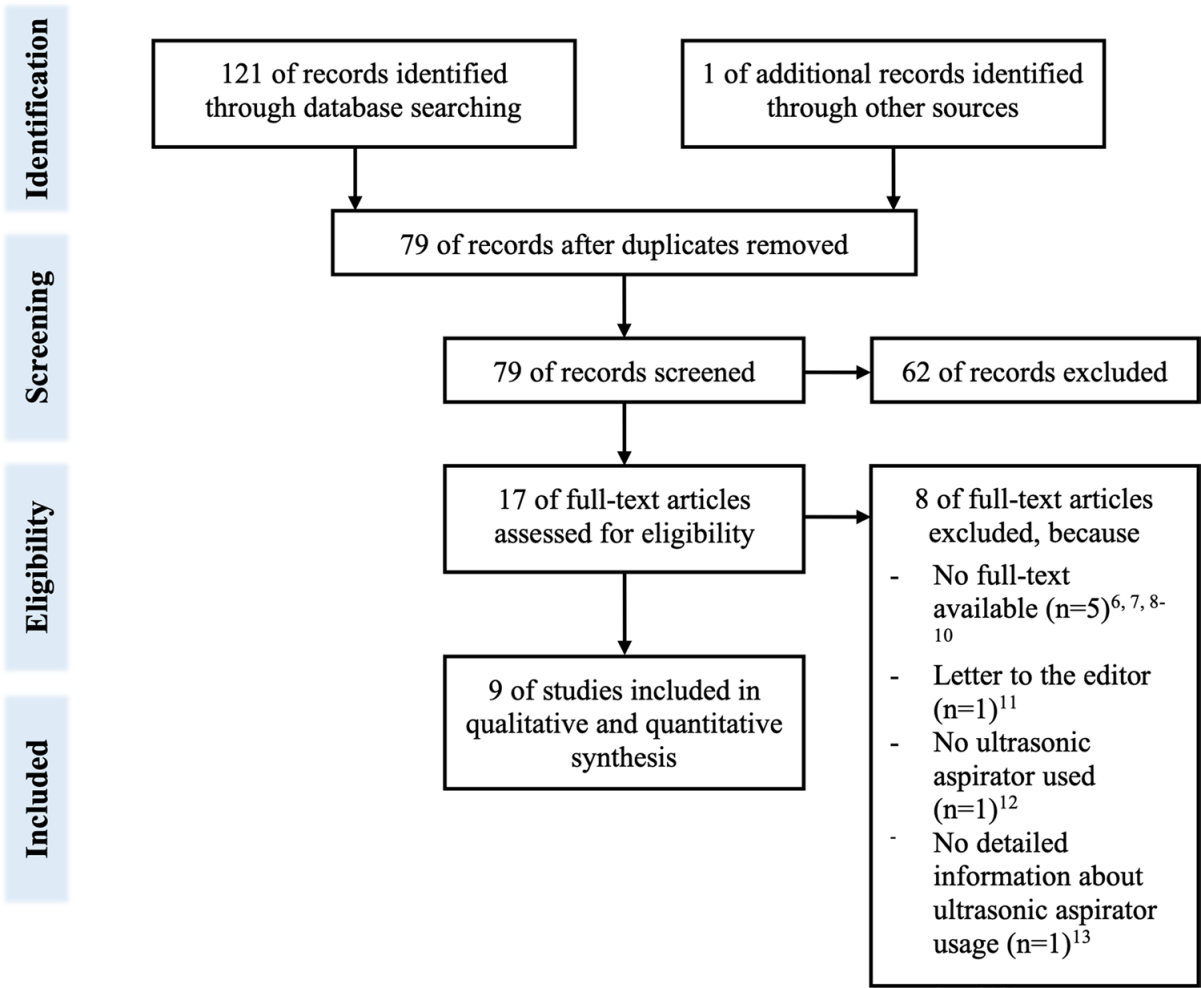
Flow chart of the number of studies identified in the systematic literature search and excluded during the analysis

A total of 121 studies were identified during the systematic literature search. Additionally, one study based on reference search was added. After excluding 43 duplicates, 79 articles were reviewed, and 62 were excluded based on the title and abstract. Of the 17 full texts reviewed, we excluded a total of eight studies due to various reasons (Fig. 2) [6–13]. This finally resulted in nine studies, which we included in the analysis (Fig. 2) [2, 14–21]. These studies consisted of 6 case series [2, 15, 18–21] and three case reports [14, 16, 17]. A total of 40 patients were analyzed in the included studies, of whom 19 (47.5%) were children (Table 1). The mean patient age was 25 years (± 23.5, range 0.42–74), and 64.9% were men.

**Table 1:** Overview of included studies with the respective included number of patients, as well as the extent of resection, clinical outcome during the last follow-up, morbidity, mortality, and recurrence rate in percent

References	No. of patients	PE (%)	GTR/NTR (%)	Clinical outcome Ns/I/W (%)	Morbidity (%)^†^	Surgery-related mortality (%)	Rec (%)	FU (m)
Cinalli et al.^18^	12	100	66.7	NA	8.3	0	33.3	10.7 ± 6.6
Ibanez et al.^19^	9	100	55.6	NA	33.3	0	0	15.1 ± 11.2
Ebel et al.^20^	8	50^‡^	100	50/50/0	50	0	0	15.9 ± 6.3
Tirado et al.^15^	3	100	66.7	0/50/0	0	0	NA	NA
Oertel et al.^2^	1*	100	NA	NA	0	0	NA	NA
Desse et al.^16^	1	100	NA	-	-	100	-	2.9
Selvanathan et al.^14^	1	100	100	NA	100	0	0	36
Gerard et al.^17^	1	100	100	0/100/0	100	0	0	6
Spennato et al.^21^	4	100	0	NA	50	0	100	34.5 ± 35.5
*Total*	*40*	*90*	*62.5*	*33/67/0*	*32.5*	*2.8*	*20*	*16.4* ± 15.5

The numbers given are in percentages

*PE* purely endoscopically, *Ns* no symptoms, *I* symptoms improved, *W* worse symptoms, *Rec* recurrence, *NA* not available, *FU* follow-up, *m* months

^*^Five patients were originally described in this case series. However, two patients underwent endonasal surgery and two patients underwent surgery for intraventricular hemorrhage using the NUA and were therefore excluded for this review

^†^This number reflects the percentage of patients who had one or more transient postoperative complications. No permanent morbidity occurred

^‡^The remaining 50% of patients underwent endoscopic-assisted surgery with the use of a microscope

### Extent of resection

Of the nine studies, seven (77.8%) reported on the extent of resection [14, 15, 17–21] (Table 1). Information about the EOR was available in 38 (95%) patients. GTR/NTR was achieved in 62.5%. No significant differences in lesion size, tumor type, and tumor location were observed between patients who underwent GTR/NTR and STR, respectively (Table 2).

**Table 2:** Overview of the different tumor characteristics in total and divided into the groups gross total/near total and subtotal resection

	No. of studies reported (%)	No. of patients (%)	GTR/NTR	STR	*p*
Lesion size (mm)	5/9 (55.6)^16,17,19–21^	24.6 ± 11.2	22.9 ± 11.1	28.8 ± 11.4	0.259
Types of tumor	9/9 (100)^2,14–21^				0.054
Colloid cyst		7 (17.5)	5 (20)	1 (7.7)	
Pilocytic astrocytoma		5 (12.5)		5 (38.5)	
SEGA		3 (7.5)	3 (12)		
Subependymoma		3 (7.5)	3 (12)		
Craniopharyngioma		3 (7.5)	2 (8)		
Low grade intraparaventricular tumor		2 (5)	1 (4)	1 (7.7)	
Meningioma		2 (5)	2 (8)		
Glioma (unclear dignity)		2 (5)	1 (4)	1 (7.7)	
Pilomyxoid astrocytoma		2 (5)		2 (15.4)	
Papillary tumor of pineal region		1 (2.5)	1 (4)		
Pineal anlage tumor		1 (2.5)	1 (4)		
Atypical plexus papilloma		1 (2.5)	1 (4)		
Neurocytoma		1 (2.5)		1 (7.7)	
Glioneuronal tumor		1 (2.5)	1 (4)		
Choroid plexus carcinoma		1 (2.5)	1 (4)		
Medulloblastoma		1 (2.5)	1 (4)		
Epidermoid		1 (2.5)		1 (7.7)	
Atypical teratoid rhabdoid tumor		1 (2.5)	1 (4)		
Teratoma		1 (2.5)		1 (7.7)	
Astrocytoma grade 2		1 (2.5)	1 (4)		
Tumor location	8/9 (88.9)^14–21^				0.267
Third ventricle		19 (47.5)	9 (36)	9 (69.2)	
Lateral ventricle		14 (35)	12 (48)	2 (15.4)	
Tectum		3 (7.5)	2 (8)	1 (7.7)	
Thalamus		2 (5)	1 (4)	1 (7.7)	
Aqueduct of sylvius		1 (2.5)	1 (4)		

*No.* number, *GTR* gross total resection, *NTR* near total resection, *STR* subtotal resection, *SEGA* subependymal giant cell astrocytoma

### Tumor characteristics

All studies included in the quantitative analysis reported on tumor type. The exact tumor location was reported by eight studies (88.9%) [14–21]. Tumor size was reported only by five studies in 23 of 40 cases (57.5%) [16, 17, 19–21]. Of the 40 tumors resected by NUA, the most frequent were colloidal cysts (17.5%), pilocytic astrocytomas (12.5%), subependymal giant cell astrocytomas (SEGA) (7.5%), subependymomas (7.5%), and craniopharyngiomas (7.5%) (Table 2). Tumor location was intraventricular in 34 cases (85%), tectal in three cases (7.5%), and thalamic in two cases (5%). The mean tumor size was 24.6 (± 11.2 mm, range 9 to 45 mm).

### Technical aspects

In all included studies, the use of the NUA from Söring (Endoscopic Neurosurgical Pen, Söring GmbH, Quickborn, Germany) was described [2, 14–21]. Eight studies (88.9%) described the endoscope used and six studies (66.7%) reported their endoscopes’ angulation [2, 15–21]. Most frequently (77.5%), the Gaab rigid endoscope (Karl Storz GmbH, Tuttlingen, Germany) was used. In four cases (10%), the InVent endoscope (Aesculap, Inc., Tuttlingen, Germany) was used alternatively (Table 3). A 0° endoscope was used in 21 cases (52.5%) and a 30° endoscope in 2 cases (5%). In 12 cases where a Gaab rigid endoscope was used, the angulation of the endoscope was not described [18].

**Table 3:** Overview of technical aspects of using the neuroendoscopic ultrasonic aspirator

	No. of studies reported (%)	No. of patients (%)
Type of NUA	9/9 (100%)^2,14–21^	
ENP (Söring GmbH)		40 (100)
Type of endoscope*	8/9 (88.9)^2,15–21^	
0° Gaab rigid		18 (45)
30° Gaab rigid		1 (2.5)
Gaab rigid (angulation not available)		12 (30)
0° InVent		3 (7.5)
30° InVent		1 (2.5)
Add. using microscope	9/9 (100)^2,14–21^	
No		36 (90)
Yes		4 (10)
Type of approach	9/9 (100)^2,14–21^	
Precoronal		29 (72.5)
Frontal hairline		6 (15)
Posterior parietal		5 (12.5)
Add. Intraoperative procedure	9/9 (100)^2,14–21^	
Septostomy		6 (15)
ETV		5 (12.5)
EVD		3 (7.5)
Rickham and ventricular catheter		1 (2.5)
Septostomy and EVD		1 (2.5)
Septostomy and ETV		1 (2.5)
Septostomy and foraminoplasty		1 (2.5)

*No.* number, *NUA* neuroendoscopic ultrasonic aspirator, *ENP* endoscopic neurosurgical pen, *Add.* additionally, *SEGA* subependymal giant cell astrocytoma, *ETV* endoscopic third ventriculostomy, *EVD* external ventricular drain

^*^In the case series by Spennato et al., it is described that in the 4 patients, either the Gaab or the InVent endoscope was used. No further specification was made

In 90% of the reported cases, a purely endoscopic procedure was performed [2, 14–19, 21]. In the remaining 10% of cases, the surgery was converted to an open microscopic procedure [20]. All studies reported the surgical access [2, 14–21]. The most common approach was a frontal approach (87.5%), either precoronal or at the frontal hairline [2, 14–16, 18–21]. In five cases (12.5%), a posterior approach was performed [17, 18, 20] (Table 3). One study by Cinalli et al. described using a thulium laser (RevoLix Jr, LISA Laser Germany) for coagulation [18]. The remaining studies used mostly monopolar electrocautery or moderate irrigation for hemostasis. In addition to resection of the lesion, intraoperative septostomy (15%), endoscopic third ventriculostomy (12.5%), and an external ventricular drainage insertion (7.5%) were performed as well (Table 3).

### Complications and follow-up

All included studies reported on perioperative complications [2, 14–21]. The most common intraoperative complications reported were bleeding (7.5%), loss of vision due to ruptured colloid cyst or air bubbles produced by the NUA (5%), and abrasion of the fornix (5%). Overall, 13 patients (32.5%) had at least one postoperative complication. The most common postoperative complications were secondary hydrocephalus (10%), meningitis/meningoencephalitis (7.5%), cognitive impairment (7.5%), and subdural hygroma (7.5%). All three cases reported with cognitive impairment were transient and completely regressed during follow-up [20]. In all cases of meningitis or meningoencephalitis, which were diagnosed based on clinical signs and altered CSF parameters, cultures remained sterile [16, 19, 20]. The patients who developed subdural hygromas and secondary hydrocephalus were treated by subduroperitoneal shunt (*n* = 2) and a ventriculoperitoneal shunt (*n* = 5), during the postoperative course [17–21]. In the included studies, no permanent morbidity was reported.

Seven of the nine studies (77.8%) described follow-up duration which averaged 16.4 ± 15.5 months [14, 16–21]. Clinical course during follow-up was reported by only 33.3% (*n* = 3) of the studies [15, 17, 20]. In 20% of the patients, preoperative symptoms improved during follow-up, and in 10% of patients, no symptoms were present. Information regarding the occurrence of recurrence was available in 66.7% of studies [14, 17–21]. In 40% of the patients, there was no recurrence. In 22.5%, the tumor residue left intraoperatively was radiologically stable, and in eight cases (20%), there was tumor progression during follow-up. Three patients died due to tumor progression 6 and 7 months after total resection of an intraventricular medulloblastoma located in the frontal horn, an atypical teratoid rhabdoid tumor of the third ventricle and after STR of a pilomyxoid astrocytoma of the third ventricle [18, 21]. One patient, who showed tumor progression of a craniopharyngioma underwent open microsurgery 6 months after neuroendoscopic STR [18]. One patient showed recurrence following totally resected pineal anlage tumor, after which he received chemotherapy [18]. Two patients are described, who underwent open microsurgical tumor resection one month after neuroendoscopic STR of an immature teratoma of the pineal region and 3 months after STR of a thalamic low-grade glioneuronal tumor, respectively [18]. In the case series of Spennato et al. all patients showed tumor progression after STR of pilomyxoid or pilocytic astrocytoma. Three of four patients underwent open microsurgical resection for tumor progression during follow-up. One patient underwent a second endoscopic tumor resection for tumor progression 8 months postoperatively [21]. Overall, one case (2.5%) reported by Desse et al. resulted in surgical mortality due to extensive meningoencephalitis after a colloid cyst ruptured intraoperatively [16] (Table 4).

**Table 4:** Overview of outcome parameters

	No. of studies reported (%)	No. of patients (%)
Surgery duration (min) mean ± SD	3/9 (33.3)^18–20^	89.4 ± 62.3
Extent of resection	7/9 (77.8)^14,15,17–21^	
Complete		20 (50)
Near complete (> 95%)		5 (12.5)
Subtotal		13 (32.5)
Intraop. complications	9/9 (100)^2,14–21^	
Hemorrhage		3 (7.5)
Loss of vision*		2 (5)
Abrasion of fornix		2 (5)
Postop. transient complications	9/9 (100)^2,14–21^	
Secondary hydrocephalus		4 (10)
Meningitis/-encephalitis		3 (7.5)
Cognitive impairment		3 (7.5)
Subdural hygroma		3 (7.5)
Nerve palsy		1 (2.5)
Clinic symptoms at follow-up	3/9 (33.3)^15,17,20^	
No symptoms		4 (10)
Improved symptoms		8 (20)
Worse		-
Follow-up duration (months) mean ± SD	7/9 (77.8)^14,16–21^	16.4 ± 15.5
Recurrence	6/9 (66.7)^14,17–21^	
No		16 (40)
Stable residual		9 (22.5)
Progression		8 (20)
Surgery related mortality^†^	9/9 (100)^2,14–21^	1 (2.5)

*No.* number, *SD* standard deviation, *Intraop.* intraoperative, *Postop.* postoperative

^*^The loss of visibility was due to rupture of the colloid cyst with egress of the contents in one case and to the formation of air bubbles by the NUA in another case

^†^A case with postoperative acute disseminated meningoencephalitis, which was fatal, was reported by Desse et al.^14^

## Discussion

Based on this systematic review, colloid cysts and pilocytic astrocytomas were most frequently resected by NUA, and GTR/NTR was obtained in the majority of cases. Permanent morbidity did not occur.

### Tumor characteristics

Selvanathan et al. was the first to describe the use of NUA for neuroendoscopic resection of solid tumors [14]. Cinalli et al. showed that the endoscopic removal of brain tumors is not limited to intraventricular tumors but can also be applied to solid tumors such as SEGAs or craniopharyngiomas that are located suprasellar or paraventricular [18]. Further, in the work of Oertel et al., NUA was successfully used for the resection of pituitary adenomas [2]. Resection of solid tumors using NUA requires careful adaptation of the aspiration and cavitation power[18]. Depending on the consistency, cavitation intensity in the case series of Ibanez et al. was between 60 and 80% for solid tumors [19]. For soft colloid cysts, the intensity was reduced to 20%[19]. Based on our experience for soft lesion such as colloid cyst, an intensity of 20–30% is sufficient and safe, while for solid lesions (e.g., meningiomas), the intensity needs to be risen to 70–80% [20]. According to the literature, the use of the NUA for neuroendoscopic tumor resection is particularly suitable for smaller (< 2 cm) and soft tumors with a poor vascularization [18, 20]. The use of the NUA is not suitable for very hard tumors such as teratomas [18]. Differences between GTR/NTR and STR in terms of lesion size, tumor type and location were not found in this study (Table 2). Out of the 13 (32.5%) patients with lesions larger than 2 cm, in seven patients a GTR could be achieved using a pure endoscopic or an endoscopic-assisted approach in 77.8% and 22.2%, respectively. Furthermore, in five (38.4%) patients with lesions larger than 2 cm transient complications, such as cognitive impairment, meningitis with consecutive secondary hydrocephalus and postoperative hygroma occurred (results not shown).

### Endoscopic versus microscopic resection of intraventricular lesions

There are different access techniques to reach intra- or paraventricular lesions. Depending on the surgical technique, whether a pure endoscopic, an endoscopic-assisted, or a microscopic resection is desired, different approaches and techniques are chosen, which are associated with different advantages and disadvantages. Pure microscopic approaches to the third or lateral ventricle can be divided into two major categories: transcortical and interhemispheric/transcallosal [22].

Milligan et al. presented a series of 127 patients with intraventricular tumors, 75 of whom were operated through a transcallosal approach and 52 through a transcortical approach. The most common lesions were colloid cysts, pilocytic astrocytomas, meningiomas, and diffuse astrocytomas. Overall, the mean tumor diameter was 3 ± 1.7 cm. GTR or NTR was achieved in 87% of cases and was comparable between the two different approaches. At least one postoperative complication occurred in 88 (69%) patients. Aphasia/abulia, cognitive impairment, hemiparesis and epileptic seizures were the most common postoperative complications. At the last follow-up, most patients recovered from the initial postoperative deficits, with 23.6% having persistent impairments [22].

A meta-analysis by Sayehmiri et al. compared microscopic and endoscopic resection of colloid cysts. They could show that microscopic resection was associated with higher GTR rates (98.15% versus 91.29%), and a lower risk of recurrence compared to endoscopic colloid cyst resection. However, the postoperative complication rate in patients with endoscopic resection was significantly lower compared to microscopic resection (10.42% versus 20.68%) [23]. A further meta-analysis showed similar results with a significantly lower morbidity rate associated with endoscopic colloid cyst resection (10.5% versus 16.3%) [24]. The most common complications in the microscopic group were cognitive impairment (5.1%), seizures (4.3%), and venous infarction (2.1%) and in the endoscopic group cognitive impairment (5%), meningitis (2.7%) and intraventricular hemorrhage (1.2%) [24].

Based on our systematic review, GTR or NTR was achieved in 62.5% using NUA. Compared to the abovementioned literature, this is below the rate of 87% achieved in microscopic resection of intraventricular lesions [22]. In contrast, transient morbidity of 32.5% found in our study, appears to be significantly lower than the 69% morbidity reported for microscopically resected intraventricular tumors [22]. One of the most frequent transient complications mentioned in our review is meningitis, although in all cases the cultures remained sterile. This could possibly indicate the presence of chemical meningitis instead of a bacterial meningitis, which has been described in the literature after neurosurgical procedures as well [25]. Despite the introduction of the NUA, which has expanded the treatment spectrum of neuroendoscopy, the options in neuroendoscopic surgery are still limited. As published in a previous study of our group, the characteristics such as size, vascularization, consistency, and architecture of the tumor can be used to estimate whether the tumor is suitable for pure neuroendoscopic resection or not [20]. Due to the limited room for maneuver during neuroendoscopic surgery, the selection of suitable tumors is essential. The advantage of neuroendoscopy is the minimal invasiveness, which, as expected, is also reflected in a reduced morbidity compared to microscopically resected tumors.

Overall, both techniques certainly have their advantages and disadvantages and depending on the factors mentioned as well as the surgeon’s experience with the respective technique the appropriate surgical approach must be carefully selected on an individual basis.

### Technical aspects

One of the technical challenges while using NUA is that intermittently, intraoperative visibility can be significantly reduced for various reasons. Air bubbles may occur secondarily due to the cavitation effect due to rapid movement [14]. This can be prevented by continuous irrigation. Cyst rupture can lead to a sudden loss of vision [16]. Furthermore, bleeding can severely restrict vision or cause a complete loss of vision. To control minor bleeding, extensive irrigation is usually sufficient. If larger vessels are involved, they can be coagulated using monopolar or laser. The problem that arises immediately when major bleeding occurs and vision is impaired is finding the source of the bleeding. In this regard, a technique described by Sufianov et al. in which the endoscope is retracted into the speculum to create a fluid-filled space between the tip of the endoscope and the bleeding site, may be used. The hemorrhage area is limited with the help of the endoscope sheath, and in conditions of the “fluid chamber” that can be intensively irrigated, the source of bleeding is adequately visualized and coagulated. This technique can help reduce blood loss and save time by identifying the source of bleeding faster [26]. Another technique is the dry field technique described by Oertel et al., in which the entire CSF is carefully aspirated to better identify the source of bleeding. Furthermore, gravity and air exposure promotes hemostasis [27]. In addition, large tumor dimensions can also obscure the view, making it difficult for the surgeon to navigate.

Most of the cases included in this review (77.5%) used the Gaab endoscope (Karl Storz GmbH, Tuttlingen, Germany) in combination with the NUA from Söring (Endoscopic Neurosurgical Pen, Söring GmbH, Quickborn, Germany) (Table 3). This endoscope was initially the only one compatible with the NUA from Söring. Two studies describe the use of the InVent endoscope (Aesculap, Inc., Tuttlingen, Germany). Gerard et al. describe adjustments were made to achieve compatibility [17]. Fortunately, the components have now been adapted so that both endoscopes, the Gaab (Karl Storz GmbH, Tuttlingen, Germany) and the InVent (Aesculap, Inc., Tuttlingen, Germany), are compatible with the NUA from Söring (Söring GmbH, Quickborn, Germany) without additional adjustments. One advantage of the InVent endoscope is the large working channel, which allows bimanual manipulation compared to the Gaab endoscope. So far, the inability to perform bimanual manipulation has been one of the major technical limitations of neuroendoscopic procedures, especially in comparison to microscopic surgery. With the InVent endoscope, this is now possible to some extent. Nevertheless, the working channel is still narrow, and the range of motion of the NUA is restricted, which is also mentioned as the main limitation of this technique by Cinalli et al. [18].

The recently published study by Tirado et al. describes for the first time a frontal endoscopic transforaminal-transchoroidal approach. By opening the taenia fornicis an extended endoscopic access to the pineal region and tectum is achieved [15]. Other technical aspects in the use of the NUA are that often in neuroendoscopic procedures two experienced surgeons are needed to control the endoscope and the different instruments. Alternatively, an endoscope holder can be used, but this requires adjustment each time the perspective is changed, which might be time-consuming.

### Future perspectives

It can be challenging to keep one’s orientation during neurosurgical operations, especially tumor resection. There are several commercially established navigation systems. Preoperatively, important structures can be color-coded and displayed intraoperatively. The microscope can also be navigated, and it is possible to have the marked structures displayed in augmented reality when looking through the microscope into the operating field. Navigating the endoscope is possible but projecting the different anatomical structures or the tumor to the endoscopy screen is currently unavailable. Augmented reality could potentially be used in neuroendoscopy in the future, making navigation easier and ultimately making minimally invasive neurosurgery safer.

Furthermore, the angled endoscopes allow us to gain insight into areas that remain hidden with the microscope. Unfortunately, there are currently no commercial ultrasound aspirators with an angled or controllable flexible tip. Since the angle of entry of the speculum limits the movement of the ultrasound aspirator, it is not always possible to reach these areas. With an angled tip of the UA, such regions could be reached better. A robotic flexible tip that can be controlled would be ideal for better maneuvering around the tumor during its resection. It would be even more effective to resect large tumors with complicated configurations with an angled or flexible tip of the ultrasound aspirator. In addition, Söring’s ultrasonic aspirator for open neurosurgical tumor resections was recently introduced with an extension for intraoperative neuromonitoring. This allows intraoperative subcortical mapping, which can significantly increase tumor resection’s safety. Unfortunately, such systems are not available for NUA yet. This would be an advantage for neuroendoscopic tumor surgery around eloquent areas such as the thalamus and other periventricular structures. Furthermore, endoscopic bipolar forceps would be very helpful, where the tips can be spread, and thus targeted coagulation of vessels would be possible. Finally, an endoscopic aspirator, with which targeted suction would be possible, would aid significantly in neuroendoscopic resection of lesions.

## Conclusion

The neuroendoscopic resection of brain tumors by NUA was designed to keep the surgical access and thus the collateral damage to the surrounding brain tissue as small as possible. Our systematic literature review showed that in 62.5% a GTR/NTR could be achieved with an overall transient morbidity rate of 32.5% and no permanent morbidity. A careful selection of the patients to be operated by NUA is essential. The instruments for neuroendoscopic tumor resection are continuously evolving, and we expect that the range of applications for NUA will increase in the future. Therefore, larger studies reporting on NUA use are essential to emphasize its safety and benefits further.

## Supplementary Information

Below is the link to the electronic supplementary material.

Supplementary file1 (DOCX 32 KB)

## Data Availability

Not applicable.
